# Evaluating Thrombolysis Rates and Emergency Department Time Targets in Acute Ischemic Stroke: Need for Personalized Medicine

**DOI:** 10.3390/jpm14090955

**Published:** 2024-09-09

**Authors:** Daian Ionel Popa, Florina Buleu, Carmen Williams, Anca Tudor, Dumitru Sutoi, Cosmin Iosif Trebuian, Covasala Constantin Ioan, Dragoș Forțofoiu, Marius Badalica-Petrescu, Ion Petre, Aida Iancu, Ovidiu Alexandru Mederle

**Affiliations:** 1 Doctoral School, Faculty of General Medicine, “Victor Babes” University of Medicine and Pharmacy, 300041 Timisoara, Romania; daian-ionel.popa@umft.ro (D.I.P.); dumitru.sutoi@umft.ro (D.S.); trebuian.cosmin@umft.ro (C.I.T.); 2 Department of Surgery, Emergency Discipline, “Victor Babes” University of Medicine and Pharmacy, 300041 Timisoara, Romania; mederle.ovidiu@umft.ro; 3 Department of Cardiology, “Victor Babes” University of Medicine and Pharmacy, 300041 Timisoara, Romania; marius.badalica-petrescu@umft.ro; 4 Emergency Municipal Clinical Hospital, 300254 Timisoara, Romania; drcarmen.williams@yahoo.com; 5 Department of Functional Sciences, “Victor Babes” University of Medicine and Pharmacy, 300041 Timisoara, Romania; atudor@umft.ro (A.T.); petre.ion@umft.ro (I.P.); 6 Emergency Clinical Hospital, 310003 Arad, Romania; covasala.constantin@gmail.com; 7 Doctoral School, University of Medicine and Pharmacy of Craiova, 200349 Craiova, Romania; fortofoiudragos@gmail.com; 8 Department of Radiology, “Victor Babes” University of Medicine and Pharmacy, 300041 Timisoara, Romania; aida.parvu@umft.ro

**Keywords:** acute care, acute ischemic stroke, emergency department time targets, rt-PA, thrombolysis, personalized medicine, patient-related factors

## Abstract

Background and objectives: In the era of personalized medicine, standard protocols regarding the management of acute ischemic stroke (AIS) focus on time targets alone without tailoring the protocol to the specific patient and hospital characteristics to increase IV thrombolysis rates and improve outcomes for these patients by considering organizational differences and patient-related factors that influence adherence to target times at the emergency department level. With this in mind, we evaluate the effect of achieving ED time targets from standard protocol and patient-related risk factors on the intravenous (IV) thrombolysis rate in patients with AIS in the therapeutic window. Materials and Methods: For our research, we enrolled people who arrived at the ED with signs of recent AIS with an onset of less than 4.5 h. Initially, 355 patients were included in the study, but through careful screening, only 258 were considered eligible to participate. Of the final group of 258 patients, only 46 received intravenous thrombolysis treatment. Results: In our study, when we are analyzing ED times in patients admitted with stroke symptoms in the therapeutic window, we found statistically significantly decreased ED times for patients that performed IV thrombolysis compared to patients not performing as follows: a median of 100 min in onset-to-ED door time (*p* < 0.001), a door-to-physician time (ED doctor) of 4 min (*p* = 0.009), door-to-blood-samples of 5 min (*p* = 0.026), a door-to-CT time of 15.5 min (*p* = 0.009), and door-to-CT results of 37 min (*p* < 0.001). In addition, patients who received intravenous thrombolysis were found to be significantly older (*p* < 0.001), with lower height and weight (*p* < 0.001 for both) and lower Glasgow Coma Scale (GCS) scores (9 ± 4.94 vs. 13.85 ± 2.41, *p* < 0.001). The logistic regression analysis indicated that the onset-to-ED time (*p* < 0.001) and the door-to-physician time (*p* = 0.014) for emergency medicine physicians are significant predictors of the likelihood of administering thrombolysis. By analyzing the impact of comorbidities, we observed that dyslipidemia, chronic arterial hypertension, and diabetes mellitus are significant predictive factors for performing IV thrombolysis (the presence of dyslipidemia and diabetes mellitus are predictive factors for performing IV thrombolysis, while the presence of arterial hypertension is not). Conclusions: The ED time targets that significantly influenced IV thrombolysis in our study were the onset-to-ED door time and the time it takes for the ED doctor to assess the AIS patient (door-to-physician time). The IV thrombolysis rate for these patients was 17.83%, lower than expected despite achieving most ED time targets, with the presence of chronic arterial hypertension as a significant predictive patient-related factor for not performing it. Even though our reported hospital’s thrombolysis rate is favorable compared to international reports, there is always room for improvement. Based on our study results, it is necessary that new protocols to customized standard protocols and ED time targets for increasing IV thrombolysis rate in patients with AIS in the therapeutic window, focusing more on patient-related factors and type of hospitals, granting personalized medicine its right. Based on our study results, it is necessary that new protocols customize standard protocols and ED time targets for increasing IV thrombolysis rate in patients with AIS in the therapeutic window, focusing more on patient-related factors and type of hospitals, granting personalized medicine its right.

## 1. Introduction

The efficiency of thrombolysis using recombinant tissue plasminogen activators (rt-PA) for acute ischemic stroke (AIS) is greatly influenced by time. It diminishes gradually, even for patients experiencing symptoms within the 4.5-h therapeutic window [1]. Even though intravenous (IV) thrombolysis has been in use for more than 20 years, its availability and implementation on a global scale are still needed [2,3]. It is truly astounding that only around 30% of countries have reported administering IV thrombolysis for acute ischemic stroke [3]. Furthermore, across various European nations, there exists a notable variation in the utilization of IV thrombolysis, with the highest rates reaching 20.6% and an overall average of 7.3% of incidents in AIS patients receiving this treatment [4]. Romania stands out as a cause for concern in Europe due to its significantly higher incidence of AIS compared to other regions [5], with increased costs generated by these patients [6] due to a national thrombolysis rate of lower than 10% despite a remarkable increase over the last 5 years from just 0.8% [7].

Therefore, hospital staff, prehospital services, or bystanders should promptly identify symptoms and signs of stroke while achieving the recommended management time, which is strongly emphasized in current guidelines and local protocols for the initial care of AIS patients [2,8]. Ensuring the rapid arrival of stroke patients to the emergency department (ED) is also crucial. The time required for patients to receive a diagnosis and be transferred between hospitals can contribute to delays during door-in-door-out transfers. However, emergency management times have also been shown to play a significant role in these cases [9]. On the other hand, delays during the door-to-needle time and impact on the IV thrombolysis rate depend mainly on ED staff, including nurses and physicians [10]. It has been given a significant responsibility to those professionals involved in the emergency care of acute stroke patients. Several studies in the literature have highlighted different obstacles to implementing guideline-based treatment for AIS patients [11,12,13]. Inadequate knowledge, lack of management skills, and lack of awareness of stroke symptoms are common obstacles faced by medical staff involved in AIS care, particularly in EDs. In addition, they have been shown to often lead to an inability to meet emergency department time targets or to an increased use of IV thrombolysis [12,14].

In treating AIS, personalized medicine tailors interventions to the specific characteristics of each patient; they aim to improve outcomes by considering variations in biomarkers, imaging findings, and patient-related factors [15]. University hospitals, often at the forefront of medical research and innovation, possess a distinct advantage in implementing personalized approaches for acute stroke care due to their access to advanced technology, specialized expertise, and ongoing staff training [16]. However, the true potential of personalized medicine in managing AIS has yet to be sufficiently studied. Patient-related factor analysis can process numerous patient data to create personalized treatment protocols. Can all these patient-related factors be used to develop new protocols that fit all health systems? Which patients need more personalized care?

To effectively minimize delays associated with IV thrombolysis in the emergency department, it is essential to establish and implement practical and effective strategies. The absence of quantitative evidence makes it difficult to determine the most effective interventions to reduce delays. Thorough documentation of the Emergency Department’s performance in meeting stroke management time targets can facilitate ongoing enhancements in its practices. Therefore, our objective is to evaluate the impact of archiving the emergency department time targets from standard protocol and patient-related risk factors on the rate of intravenous thrombolysis in patients with AIS in the therapeutic window (within 4.5 h from onset). This evaluation will provide a theoretical basis for future research that can propose practical personalized interventions to increase the quality of acute management care in AIS patients admitted to similarly organized hospitals. 

## 2. Material and Methods

### 2.1. Study Design and Patient Population

This study enrolled patients who presented to the ED of the County Emergency Hospital from Arad, Romania, between 1 January 2020 and 31 December 2023, with symptoms of AIS. The hospital is the largest medical unit in the county and serves over 70,000 patients annually. It is classified as Category II according to the Romanian Ministry of Health. This hospital serves the county’s population in its administrative-territorial area, as well as in neighboring counties, with a high level of equipment and staffing of human resources, which ensures the provision of high-quality medical services of high complexity.

**Figure 1:**
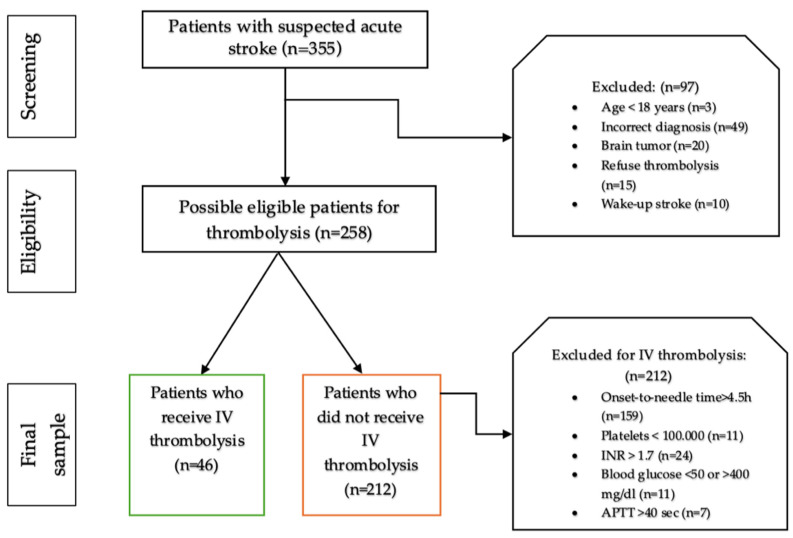
Our study flowchart.

We included in this study all consecutive patients who addressed the emergency department with symptoms of acute stroke, were identified as patients with suspected acute stroke by the triage nurse, and had complete medical records, both in electronic and paper format. Out of the initial sample of 355 patients, only 258 met the eligibility criteria for our study after careful screening. According to our national protocol, initiating intravenous thrombolysis with rt-PA is recommended within 4.5 h of the onset of stroke symptoms (“onset-to-needle time”) [8]. Therefore, patients who did not meet the eligibility criteria for intravenous thrombolysis were excluded. This involved patients under the age of 18, those diagnosed with intracerebral hemorrhage or brain tumor at the brain and neck computer tomography, and individuals who arrived at the emergency department more than 4.5 h after experiencing stroke symptoms. Both physicians the emergency medicine physician and the on-duty neurologist, decided these exclusions based on the patient’s medical history, laboratory, and head and neck CT scan results. Of the 258 remaining in the final sample, only 46 benefited from treatment with intravenous Alteplase (see Figure 1). Informed consent was obtained from all patients involved in the study.

### 2.2. Data Collection

Upon arrival at the emergency department, the medical team carefully documented the exact time of the initial onset of stroke symptoms and the mode and time of the patient’s arrival. Patients or their family members provided details of the first onset of stroke symptoms, which was recorded as the “onset time”. In cases where patients experienced symptoms while asleep, onset was determined as the last time they were free of stroke-related symptoms. Cases where patients were awakened with stroke symptoms that were not present before falling asleep were categorized as “wake-up stroke”.

**Figure 2:**
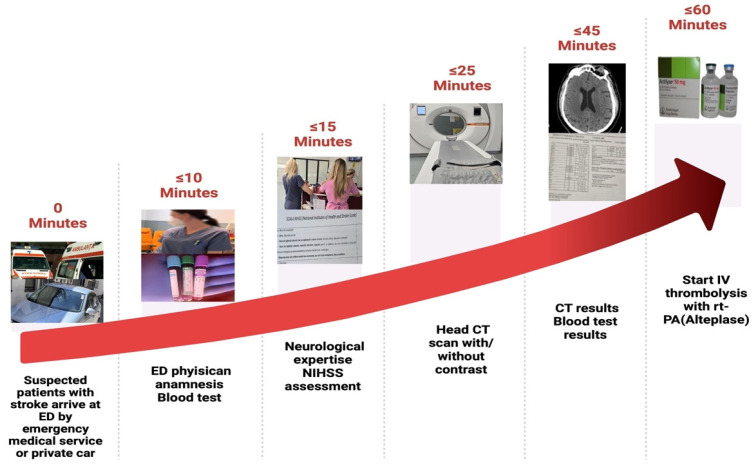
The emergency department time targets in acute ischemic stroke management [8] (created with BioRender.com).

Our national protocol [8] suggests the following ED time targets for managing AIS: onset-to-needle time should be ≤4.5 h; door-to-physician time should be ≤10 min (which includes an initial assessment by both the emergency medicine physician and neurologist, covering the last known well time, IV thrombolysis eligibility, and stroke severity evaluation); door-to-CT time should be ≤25 min; door-to-CT results should be ≤45 min; and door-to-needle time should be ≤60 min, as indicated in Figure 2.

Upon entering the emergency department, every patient promptly receives a comprehensive medical evaluation, which includes a brain computed tomography scan, with or without contrast, alongside clinical laboratory assessments such as complete blood count (CBC), international normalized ratio (INR), prothrombin time, partial prothrombin time, blood glucose, and electrolyte tests. It is essential to mention that this evaluation did not include those without medical records. The patient-related factors we considered were risk factors and comorbidities like age, gender, body mass index, obesity, hypertension, diabetes mellitus, dyslipidemia, smoking habits, chronic heart disease, chronic obstructive pulmonary disease, and chronic kidney disease.

In conjunction with the neurologist and the emergency medicine physician, the radiologist collaboratively determines the subtype, severity, and localization of the stroke by analyzing the brain imaging results and clinical findings, adhering to the stroke definition set forth by the World Health Organization in 1970, which continues to be in use today [17].

During initial clinical assessments of patients in ED, the on-duty neurologist assessed neurologic deficits and stroke severity using the National Institutes of Health Stroke Scale (NIHSS) to categorize them at different time intervals: on admission and after 1 h, 2 h, and 24 h [18].

To identify early signs of ischemia on brain CT scans, specifically in patients suspected of anterior circulation occlusion, the Alberta Stroke Program Early CT score (ASPECTS) was utilized as a screening tool to determine eligibility for reperfusion therapy. ASPECTS is a quantitative score ranging from 0 to 10, with 1 point deducted for each region showing evidence of early ischemic change—a score of 0 shows infarction in all 10 areas. A low ASPECTS score (<7) suggests larger infarctions, which are related to greater stroke severity and a higher risk of hemorrhagic transformation [19].

### 2.3. Statistical Analysis

Continuous variables were described through mean and standard deviation or median and interquartile range (IQR) values, whereas categorical variables were reported using frequencies and percentages. The Shapiro–Wilk test was used to evaluate the distribution of continuous variables. Unpaired t-tests, Mann–Whitney U tests, and Chi-square tests were applied to compare the characteristics of patients who underwent thrombolysis with those who did not. Multiple logistic regression models were utilized to identify the independent factors linked to the administration of thrombolysis.

The results were presented as odds ratios with corresponding 95% confidence intervals. A *p*-value of less than 0.05 was used to establish statistical significance. For data analysis, JASP v0.18.3, an open-source software supported by the University of Amsterdam and freely accessible, was employed.

## 3. Results

### 3.1. Baseline Characteristics of Patients at Admission to ED

The final sample consisted of 258 patients with acute stroke, from which 212 patients (82.17%) were not performing IV thrombolysis and with only 46 patients (17.83%) receiving. After dividing them into two subgroups, the following results were obtained using IV thrombolysis. Concerning gender in our study, 23 (50%) of the thrombolysis patients were male compared to 119 (56.1%) of the non-thrombolysis patients, but with statistically insignificant differences (Chi2 test, *p* = 0.449). Patients that received IV thrombolysis were statistically significantly older (*p* < 0.001), with a lower height and weight (*p* < 0.001, for both) and with a lower GCS (9 ± 4.94 vs. 13.85 ± 2.41, *p* < 0.001), as shown in Table 1.

No statistically significant differences were observed between groups regarding platelet count (*p* = 0.944), blood glucose (*p* = 0.359), and INR (*p* = 0.672). A lower hemoglobin value (*p* = 0.007), partial thromboplastin time (*p* = 0.020), and prothrombin time (*p* = 0.006) were observed in patients who received IV thrombolysis (Table 2).

### 3.2. Analysis of Correlations between Stroke Severity Scores and Performing IV Thrombolysis

The values of ASPECTS at 24 h were significantly lower in patients without performing IV thrombolysis, and ASPECTS was not statistically significantly different between groups at admission (Mann–Whitney test, *p* = 0.002 and *p* = 0.901, respectively). NIHSS values at admission, at 1 h, 2 h, and 24 h, are not statistically significantly modified between groups (Mann–Whitney test, *p* = 0.654, *p* = 0.861, *p* = 0.889, and *p* = 0.649, respectively) (Table 3).

### 3.3. Analysis of Correlations between ED Time Targets and Performing IV Thrombolysis

**Figure 3:**
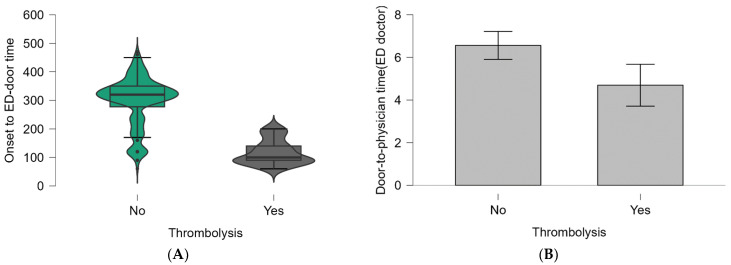
Graphical representation of the values of ED time targets (minutes) and the two groups: (**A**) The rainclouds for the onset-to-ED-door time (*p* < 0.001). (**B**) The boxplots for the door-to-physician time (ED doctor) (*p* = 0.009). Within the violin boxplots are the median and interquartile ranges.

**Figure 4:**
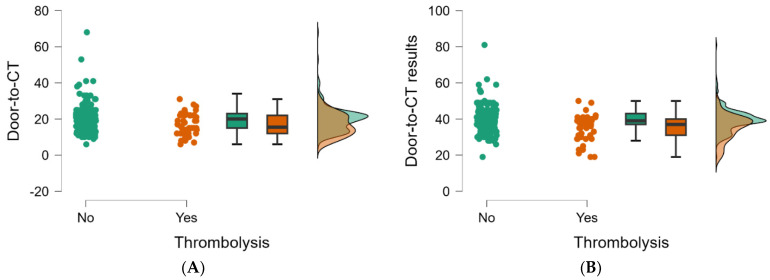
Raincloud plots of the values of ED time targets (minutes) between the two groups of patients: (**A**) The door-to-CT time (*p* = 0.009). (**B**) The door-to-CT results time (*p* < 0.001).

The examination of time targets in the emergency department for the management of AIS patients revealed that the door-to-physician (neurologist) times were 7 min (IQR, 5–10 min) for those who did not receive IV thrombolysis and 8 min (IQR, 5–9 min) for those who did. This difference was insignificant in IV thrombolysis status (*p* = 0.786). All other times measured had significant differences between studied groups (Table 4; Figure 3 and Figure 4).

Figure 3 and Figure 4 show the baseline parameters analyzed in Table 4, which decreased significantly in patients performing IV thrombolysis.

### 3.4. The Logistic Regression Analysis of ED Times Targets and Comorbidities

The logistic regression analysis (using the Enter method), where the dependent variable was IV thrombolysis, revealed that among the ED time targets, the onset-to-ED time (*p* < 0.001) and door-to-physician time (emergency medicine doctor) (*p* = 0.014) serve as predictors for the probability of performing thrombolysis. We showed that shorter durations for these two variables have a significantly higher chance of thrombolysis in patients with AIS (Table 5).

The logistic regression that analyzes the presence of comorbidities showed that dyslipidemia, chronic arterial hypertension, and diabetes mellitus are significant predictive factors for performing IV thrombolysis (the presence of dyslipidemia and diabetes mellitus are predictive factors for performing IV thrombolysis, while the presence of arterial hypertension is not) (Table 6).

## 4. Discussion

It is widely recognized that successful recanalization with IV rt-PA is crucial for achieving better clinical outcomes, depending on the therapeutic window [5]. Those who receive prompt treatment experience a 32% relative increase in their chances of having minimal or no disability after 90 days [20] or good outcomes even after 7 years [21]. Due to the critical nature of prompt intervention, protocols were released addressing different points of the treatment process. These protocols stipulate that the evaluation from door-to-physician time should take less than 10 min, the initiation of CT/MRI should occur within 25 min, and the door-to-needle time must be under 60 min [2,8]. The ED time targets analyzed in our study served as the basis for all these assessments. However, to understand the full significance of the results of this study, a thorough comparison with previous studies might provide additional insight.

In our study, when we are analyzing ED times in patients admitted with stroke symptoms in the therapeutic window, we found significantly decreased ED times for patients that performed IV thrombolysis compared to patients not performing as follows: a median of onset-to-ED door time of 100 min (*p* < 0.001), a door-to-physician time (ED doctor) of 4 min (*p* = 0.009), door-to-blood-samples of 5 min (*p* = 0.026), a door-to-CT time of 15.5 min (*p* = 0.009), and door-to-CT results of 37 min (*p* < 0.001). Only a door-to-physician time (neurologist) of 7 min (IQR, 5–10 min) for patients not performing IV thrombolysis and 8 min (IQR, 5–9 min) for patients who received IV thrombolysis was not significantly correlated (*p* = 0.786) with performing or not performing IV thrombolysis, results that highlight again the importance of ED time target compliance and the significant responsibility of ED medical staff in the management of AIS patients. The findings of our study align with research conducted in an emergency department in Iran. That study revealed that the average duration of different pre and in-hospital times, including patient arrival, initial assessment by an emergency medicine resident, presence of a neurology resident in the ED, activation of the acute stroke team, and interpretation of the CT scan, was reduced for patients who met the criteria for intravenous thrombolysis, in contrast to those who no longer qualified for fibrinolytic therapy [22]. A study focusing exclusively on emergency department patients who underwent intravenous thrombolysis found that only 88% (n = 23) received intravenous rt-PA within the recommended 60 min interval, with a mean treatment duration of 44.5 min. The mean time from arrival to a physician visit in the emergency department was 1.81 min. All patients underwent a computed tomography scan within 28 min of arrival, averaging 5.08 min. The neurologist’s on-call evaluation was initiated within 59 min of the presentation, with a mean time of 25.19 min [23]. In examining the possible explanations for the differences in ED time targets noted between the groups in our study, it is crucial to consider a range of factors that could impact these discrepancies. While this study did not delve deeply into these explanations, understanding them could offer important insights for clinical practice. For example, elderly patients or patients with multiple comorbidities frequently require more comprehensive assessments, resulting in longer time spent in the emergency department. Variations in these times could be partly attributable to differences in age distribution between groups. The impact of available and accessible diagnostic tools, including imaging and laboratory services, was significant on ED workflow, as was the delay in receiving test results. Because this study did not analyze the time required to perform thrombolysis, we argue that the neurologist’s decision did not influence other recorded ED times. When a patient was presented with stroke symptoms, the medical staff followed the protocol regardless of whether rt-PA was administered or not. All these mentioned aspects will be discussed in the following paragraphs.

Unexpectedly, our analysis demonstrated that the rate of IV thrombolysis was significantly higher than the national one reported (17.83% in our study vs. 5.4% national average) [7]. A possible explanation is that this hospital is much better equipped compared to other hospitals in the country that perform IV thrombolysis (also with availability and interpretation of CT scans 24/7, which serves only the ED and the rapid performance of laboratory tests), with an advanced infrastructure and a professional medical team that focuses on compliance with the national protocol, as well as the fact that in this study, only patients with AIS in the therapeutic window were included. Another possible reason would be the fact that there are hospitals included in our national list that perform IV thrombolysis but have a rate of 0%, such as the Emergency County Hospital in Tulcea compared with the highest rate reported by the Brasov County Emergency Hospital (almost 38% for 2023 [7]). 

In addition, after we performed logistic regression analysis considering the IV thrombolysis as the dependent variable, we observed that, from the ED time, only the onset-to-ED time (*p* < 0.001) and door-to-physician (emergency medicine physicians) time (*p* = 0.014) are predictors for performing thrombolysis; the shorter the times for these two variables are, the more significantly increased the chance of thrombolysis in patients with AIS. Upon reviewing the analysis conducted by Ganti et al., it becomes evident that certain factors significantly contribute to delays in administering thrombolysis in the emergency department. These factors include arriving during the night shift, the absence of a specialized stroke team, extended wait times for CT results, and presenting as a walk-in patient [24]. So, addressing these factors from an operational perspective is crucial when implementing quality improvement measures for hospital protocols. The findings of Baskar et al. support the use of multi-system interventions to reduce in-hospital AIS time metrics [25].

The diagnosis, initial treatment, and long-term results in stroke management can be affected by implicit bias, such as several patient-related factors like sex, age, race, and socioeconomic status. Notably, the age of the patient plays a significant role in misdiagnosis during early medical interactions, and younger individuals frequently experience misdiagnosis of stroke in emergency departments [26]. A recent meta-analysis involving almost 16,000 patients revealed that approximately 9% of all strokes were initially overlooked in the emergency department, all being young patients [27]. When comparing the two groups, we observed that patients who received IV thrombolysis were significantly older (*p* < 0.001), with a mean age of 67 years (IQR 60.75–75) of the patients who had not received rt-PA vs. 76.5 years (IQR 70–81) of the patients that performed IV thrombolysis. Maybe it is because unusual symptoms and manifestations of stroke are more frequently seen in younger individuals [26], which adds to the uncertainty in diagnosis and can lead to delays in treatment. Concerning gender in our study, 23 (50%) of the thrombolysis patients were male compared to 119 (56.1%) of the non-thrombolysis patients, but with statistically insignificant differences (Chi2 test, *p* = 0.449). The socio-demographic information of patients, in conjunction with NIHSS scores, appears to have little effect on delayed treatment [28]. We could not analyze factors like race and socioeconomic status because this is a retrospective study that is not mentioned in medical records. We can only conclude from our experience that there was no difference in the acute management of patients with AIS regarding socioeconomic status after ED admission (all patients received the same quality of ED management), and that all patients included in our study were Caucasian. Based on these biases, numerous studies have examined various factors that may affect the likelihood of benefiting from IV thrombolysis in cases of AIS. However, it has been observed that many of these factors are interconnected, and their impact can vary depending on the healthcare system performance and the social, cultural, behavioral, and economic characteristics of the population being studied [29,30,31,32]. Even in these situations, according to Botelho et al., various organizational factors and strategies can be implemented to decrease time delays and increase the number of AIS patients receiving IV thrombolysis, regardless of the situation. While most of these factors and strategies can be applied in any context, some are only effective in specific contexts [30].

The logistic regression that analyzes the impact of comorbidities in our study showed that dyslipidemia, chronic arterial hypertension, and diabetes mellitus are significant predictive factors for performing IV thrombolysis. The presence of dyslipidemia (*p* = 0.013) and diabetes mellitus (*p* < 0.001) is a predictive factor for performing IV thrombolysis, while the presence of arterial hypertension is not (*p* < 0.001). The influence of antihypertensive medications was evident in blood pressure levels at the time of ED admission, as there were no notable differences in SBP and DBP values between the two groups. However, this finding did not hold in the logistic regression analysis, where chronic hypertension was linked to reduced chances of receiving thrombolysis. We acknowledge the potential that blood pressure fluctuations in our study may be related to hemorrhagic stroke and delayed cerebral reperfusion. The literature also noted aspects regarding IV thrombolysis about hypertension. Patients who did not receive antihypertensive treatment before rt-PA experienced shorter door-to-needle times, averaging 52.6 min compared to 62.1 min (*p* = 0.016) [33]. Elevated prehospital blood pressure was also linked to extended door-to-needle times, and emergency department durations continue to be lengthy if prehospital blood pressure of 185/110 or higher is not addressed before arriving at the ED [34].

In addition, the current study has several strengths. The previous studies that investigated IV thrombolysis in ED that focused and analyzed different protocols—like establishing an emergency stroke nurse role and refining the green channel stroke process to enhance the IV thrombolysis rates for stroke patients upon their arrival at the ED—found that these measures did not significantly elevate the IV thrombolysis rates for stroke patients across various organizational emergency departments globally [35,36]. The protocol behind the HASTE project, consisting of three phases, identified weaknesses in managing these patients during ED hospitalization (HASTE I) and developed ED time-reduction strategies to improve the administration of IV thrombolysis, bringing evidence regarding the importance of identification of ED time targets not archived in acute management. Also, the correction of these times has been shown to improve cerebral reperfusion rates [37] in a systematic review by Leite et al. [38], based on studies that extensively examined the response times for suspected acute ischemic stroke. Implementing protocols and reorganizing services to treat these cases were identified as effectiveness indicators [39]. The findings revealed that hospitals that adopted a protocol experienced shorter response times. However, there is still a need to enhance the awareness of stroke symptoms among individuals who are in initial contact with the affected person and among the healthcare professionals involved in pre- and in-hospital care. It is important to note a constraint when interpreting the findings of this study’s results. They only compared two time points (pre- and post-protocol implementation) without examining cause and effect. As a result, the assessment of the included articles did not involve the tool’s inquiries on identifying confounding variables and implementing strategies to reduce loss to follow-up.

Furthermore, it underscored the necessity for studies that identify weak points to effectively implement practical interventions to enhance the quality of care for patients with AIS. For these reasons, our study analyzed factors like ED time targets that can be measured and corrected as barriers to a decreased rate of IV thrombolysis for these critical patients, together with patient-related factors. These ED time targets are easy to follow and adaptable to the available resources and patient characteristics. Also, in our study, the analysis of these ED time targets was initiated and continued in a university hospital where staff are trained to respond fast. This could be another added value of the current study. Standardized ED time target protocols can help mitigate disparities in care. For example, evidence suggests that certain racial and ethnic groups receive less timely care in emergencies. Standardized ED time targets ensure that all patients are treated based on clinical urgency rather than other factors [40] as a result of various emergency department initiatives and diverse efforts that have led to significant improvements in patient outcomes for conditions such as stroke and myocardial infarction, where timely intervention during acute care is crucial for successful outcomes [41,42]. Our study data showed that even if these standard ED time targets were followed, due to not focusing more on patient-related factors (such as chronic arterial hypertension, dyslipidemia, and diabetes mellitus), the IV thrombolysis rate remained low.

In addition, personalized stroke programs must also be performance-evaluated at the ED level to identify which areas need improvement. However, the variation in emergency medical service systems between countries and even within regions makes it difficult to replicate experiences from other places [43]. On the other hand, interventions aimed at reducing in-hospital delays compared with prehospital delays have shown more progress and have been more successful in developed areas or university hospitals [43]. One possible reason for this is the implementation of national protocols, like ours [8], which enables the monitoring of therapeutic actions in IV thrombolysis and educates hospital staff on improving their healthcare systems by reducing delays in the emergency department. Implementing advanced stroke care protocols in non-university hospitals or less-equipped facilities presents distinct challenges, yet it remains essential for improving outcomes across various healthcare environments. The implications for clinical practice in these settings highlight the necessity for adaptation, resource optimization, and strategic collaboration [44]. Numerous factors affect the design, implementation, and effectiveness of interventions based on personalized stroke programs, including, but not limited to, population size and confounding structural or systemic elements such as cultural and behavioral influences. By employing quality-improvement strategies grounded in the principles of implementation signals and enhancing health systems across the emergency care of AIS, it is feasible to improve process metrics, encourage multidisciplinary collaboration, and enhance patient outcomes [45].

Therefore, the implications of our study for medical practice are significant, particularly in terms of creating awareness about the importance of timely intervention for patients with acute ischemic stroke during ED management. By examining the ED time targets, this study has the potential to improve patient management in hospitals like ours, located in countries where stroke incidence and mortality are at an alarmingly high level. By identifying areas that need improvement, we can optimize protocols in the emergency department and positively impact stroke care at both national and international levels. It is important to note that this study focused on the therapeutic window for AIS treatment and identified weaknesses that can be addressed to enhance the thrombolysis procedure during emergency department management. Since the health system in our country recommends a therapeutic window of 4.5 h for IV thrombolysis [8], especially with rt-PA, we focused on this study only on this critical parameter in the treatment of acute ischemic stroke. This window is based on a balance between maximizing treatment efficacy and minimizing the risk of adverse outcomes, especially early hemorrhagic transformation [32]. The effectiveness and safety of IV thrombolysis with rt-PA in AIS are heavily dependent on time, making the narrow therapeutic window and time delays significant barriers to the widespread use of this treatment [46], even in nations that boast well-structured stroke networks, less than half of acute ischemic stroke instances present within the 4.5 h window. Moreover, fewer than 60% of those in the therapeutic window are administered IV thrombolysis [47].

In summary, despite extensive efforts from our team, management, and government to improve stroke care, this study reveals that almost 73% of acute ischemic stroke patients admitted to our emergency department in the therapeutic window were suitable candidates for thrombolytic therapy. So, we found that the problem of in-hospital delay during ED admission in patients with acute ischemic stroke in our hospital was quite severe, resulting in a low rate of intravenous thrombolysis; only 17.83% (n = 46) of these patients received intravenous rt-PA. The primary cause of delay in receiving care was the delayed presentation to the ED. However, our ED and acute stroke team consistently met ED time targets for eligible patients. Because of this, studies such as Extending the Time for Thrombolysis in Emergency Neurological Deficits (EXTEND) have analyzed the benefits of rt-PA administration even after the guideline recommended 4.5 h. Thus, in this randomized trial, patients with AIS who presented within 4.5–9 h of symptom onset received IV thrombolysis or placebo. At 3 months, a statistically insignificant increase was observed in symptomatic intracerebral hemorrhage (6.2% with rt-PA versus 0.9% with placebo), increases that did not affect mortality rates and resulted in a higher percentage of patients with no or minor neurologic deficits than the use of placebo. However, they conclude that this extension of the therapeutic time window may be feasible only by using advanced cerebral imaging [48]. However, it is essential to note that under 2% of consecutive AIS patients will likely meet the EXTEND clinical and neuroimaging criteria for IV thrombolysis [49].

Conversely, performance was lower for patients not eligible for fibrinolytic therapy. These findings underscore the urgent need for mechanical thrombectomy services at our institution. The majority of stroke patients within the therapeutic window did not receive intravenous rt-PA due to time constraints, thrombolysis contraindications, or other patient-related factors.

Consequently, hospitals require the implementation of mechanical thrombectomy services. This approach extends the eligibility for recanalization therapy to 24 h in specific patients, proving particularly beneficial for those with large vessel occlusions and high NIHSS scores, or for patients unable to undergo IV thrombolysis [50]. These findings highlight the necessity of an integrated multidisciplinary approach, which involves incorporating endovascular providers and culture promotion specialists into our existing team to enhance the quality of care. Also, supporting the need for an integrated multidisciplinary approach, the addition of an interventional radiologist, and focusing on reducing ED times for all patients admitted with AIS in the therapeutic window are changes that can be made to increase rates of reperfusion therapy and improve outcomes for these critical patients [32,51].

***Study limitations.*** Although this study highlights the essential components of acute stroke management in the ED, it has limitations. The most considerable and undoubtedly most critical restriction derives from the fact that the data were retrospectively collected from electronic and paper medical records. Consequently, the reliability of these findings depends on the assumption that the documentation is complete and accurate. In addition, it should be noted that findings from a study conducted in a single center may only sometimes apply to other contexts. Variations in hospital procedures, patient characteristics, and the healthcare systems in different regions can influence the relevance of the results to larger populations.

Moreover, an experienced neurologist was permanently part of our stroke team, and the decision of performing or not performing IV thrombolysis on a patient is made faster together with the emergency physician and radiologist. In our study, a neurologist was present on-site during acute stroke management in the ED, but this is most likely a privilege of a university hospital. Thus, at this point, only some of these results can be generalized to most hospitals worldwide due to the different organizations and local protocols. Another limitation to consider when interpreting the results of this study is the challenge of assessing how the acute management of our patients was prioritized compared to others simultaneously admitted to the emergency department, as this may impact the target times for AIS patients. In addition, while this study focused exclusively on patients eligible for thrombolysis in therapeutic windows, eliminating the likelihood that subsequent test results or comorbid conditions could affect ED time targets and subsequent IV thrombolysis administration, we could not account for every potential influencing factor. However, the observational design of this study leaves the possibility of residual bias, such as race, ethnicity, gender, age, or socioeconomic status.

***Recommendations.*** While the existing structure of standardized protocols in AIS management has significant value, it frequently ignores the individual differences of critically ill patients. Personalized medicine, which modifies management strategies according to patient-related factors, represents a crucial development in acute stroke management. We suggest implementing personalized stroke protocols to explore further the factors that could delay the treatment time and implicitly lead to the failure to perform IV thrombolysis, which is time-dependent. These protocols should incorporate ED time targets, as illustrated in Figure 2, while being personalized to specific hospital needs (e.g., reducing door-to-CT time in units where CT/MRI are ED exclusive or minimizing door-to-blood sample result time in EDs capable of performing rapid coagulation tests, etc.) and patient-related factors. Also, the results of this research could improve acute stroke care in emergency departments, especially those like Romania. This developing nation faces one of the highest stroke occurrence and mortality rates in the region.

## 5. Conclusions

From the ED time targets, the onset-to-ED door time and the time it takes for the patient to be assessed by an ED doctor (door-to-physician time) are essential factors in determining whether IV thrombolysis is performed in acute ischemic stroke patients who are admitted to the therapeutic window. Overall, the IV thrombolysis rate for these patients was 17.83%, lower than expected despite achieving most of the ED time targets with chronic arterial hypertension as a significant predictive patient-related factor for not performing it.

In AIS, conventional medical practices have frequently adopted a one-size-fits-all strategy for treatment, presuming that identical ED time targets apply universally. Nevertheless, from our study results, it is becoming more evident that factors related to patients significantly influence these outcomes. Our findings have sparked the rise of personalized protocol—an approach that customizes medical interventions based on individual patients’ distinct characteristics and the health system organization.

## Figures and Tables

**Table 1:** Descriptive data of analyzed patients according to performing or not performing IV thrombolysis.

Variable	Thrombolysis	Valid	Mean ± SD	Median (Q1–Q2)	*p*
Age, years	No	212	66.71 ± 11.94	67 (60.75–75)	<0.001 *
	Yes	46	74.39 ± 10.94	76.5 (70–81)	
Male, n	No	119 (56.1%)	-	-	0.449
	Yes	23 (50%)	-	-	
Height, cm	No	212	172.11 ± 7.9	172 (167–178)	<0.001 *
	Yes	46	163.28 ± 4.49	165 (160–165)	
Weight, kg	No	212	78.03 ± 12.32	80 (70–85)	<0.001 *
	Yes	46	70.96 ± 10.42	70 (65.75–77.25)	
SBP, mmHg	No	212	154.54 ± 20.57	154.5 (140–170)	0.392
	Yes	46	155.98 ± 21.31	160 (141.25–175)	
DBP, mmHg	No	212	81.46 ± 12.26	80 (75–90)	0.817
	Yes	46	81.2 ± 15.92	80 (70–93.75)	
GCS	No	212	13.85 ± 2.41	15 (14–15)	<0.001 *
	Yes	46	9 ± 4.94	10 (3.25–15)	

* Significant difference. SBP, systolic blood pressure; DBP, diastolic blood pressure; GCS, Coma Glasgow Score; values are expressed as means ± standard deviation (SD) or by median (interquartile range).

**Table 2:** Blood test sample results between groups at admission in the ED.

Variable	Thrombolysis	Valid	Mean ± SD	Median (IQR)	*p*
Platelets count, ×10^9^ uL	No	212	220.63 ± 63.45	219 (171.75–255)	0.944
	Yes	46	228.35 ± 90.57	221 (176–248.5)	
Hemoglobin, mg/dL	No	212	13.56 ± 1.85	14 (12–15)	0.007 *
	Yes	46	12.85 ± 1.55	13 (12–14)	
Blood Glucose, mg/dL	No	212	140.32 ± 48.49	125.5 (104–171.25)	0.359
	Yes	46	130.83 ± 37.69	121 (104.25–150.75)	
INR	No	212	1.68 ± 1.72	1.18 (1.038–1.41)	0.672
	Yes	46	1.27 ± 0.36	1.225 (1.08–1.39)	
Partial thromboplastin time, sec	No	212	29.3 ± 15.32	25.85 (23.075–29.225)	0.020 *
	Yes	46	28.37 ± 4.91	28.1 (24.8–31.3)	
Prothrombin time, sec	No	212	17.65 ± 19.63	12.95 (12–14.9)	0.006 *
	Yes	46	14.69 ± 2.83	14.3 (13.025–15.25)	

* Significant difference. INR is the international normalized ratio. Values are expressed as means ± standard deviation (SD) or by median (interquartile range).

**Table 3:** Stroke severity scores between both groups.

Variable	Thrombolysis	Valid	Mean ± SD	Median (IQR)	*p*
NIHSS at presentation	No	212	14.49 ± 5.33	15 (10–19)	0.654
	Yes	46	13.94 ± 6.15	15 (8.5–19)	
NIHSS at 1 h	No	212	12.3 ± 6.21	13 (7–17)	0.861
	Yes	46	12 ± 6.86	11.5 (6–18)	
NIHSS at 2 h	No	212	11.37 ± 6.48	12 (6–16)	0.889
	Yes	46	11.48 ± 7.01	10 (4.5–17.75)	
NIHSS at 24 h	No	210	10.65 ± 7.01	10 (5–16)	0.649
	Yes	45	11.33 ± 8.32	9 (3.25–18.75)	
ASPECTS	No	212	9.43 ± 0.86	10 (9–10)	0.901
	Yes	46	9.5 ± 0.69	10 (9–10)	
ASPECTS at 24 h	No	210	7.68 ± 1.59	8 (7–9)	0.002 *
	Yes	45	8.48 ± 1.23	9 (7–9)	

* Significant difference. Values are expressed as means ± standard deviation (SD) or by median (interquartile range).

**Table 4:** ED time targets between the two groups.

Variable	Thrombolysis	Valid	Mean ± SD	Median (IQR)	*p*
Onset-to-ED door	No	212	298.63 ± 86.26	320 (277.5−350)	<0.001 *
	Yes	46	116.74 ± 42.69	100 (90−140)	
Door-to-physician (ED doctor)	No	212	6.56 ± 4.85	5 (3−9)	0.009 *
	Yes	46	4.7 ± 3.31	4 (2.25−6)	
Door-to-physician (Neurologist)	No	212	7.93 ± 4.88	7 (5−10)	0.786
	Yes	46	7.52 ± 3.31	8 (5−9)	
Door-to-blood samples	No	212	8.14 ± 2.42	10 (5−10)	0.026 *
	Yes	46	7.28 ± 2.73	5 (5−10)	
Door-to-CT	No	212	19.92 ± 7.33	20 (15−23)	0.009 *
	Yes	46	16.72 ± 6.13	15.5 (12−22)	
Door-to-CT results	No	212	39.72 ± 6.68	39 (37−43)	<0.001 *
	Yes	46	35.26 ± 7.15	37 (31−40)	

* Significant difference. Values are expressed as means ± standard deviation (SD) or by median (interquartile range). The ED times are expressed in minutes (min).

**Table 5:** Logistic regression considers thrombolysis as a dependent variable and ED times as an independent variable.

Variables in the Equation	B	S.E.	Wald	df	Sig.	Exp(B)	95% C.I. for EXP(B)	
							Lower	Upper
Onset-to-door	−0.232	0.017	31.105	1	<0.001 *	0.937	0.927	0.970
Door-to-physician (ED doctor)	−0.508	0.209	5.823	1	0.014 *	0.506	0.450	0.941
Door-to-physician (neurologist)	0.119	0.125	2.047	1	0.194	1.215	0.945	1.998
Door-to-blood samples	−0.026	0.124	0.044	1	0.782	0.955	0.431	1.570
Door-to-CT	−0.194	0.025	2.441	1	0.103	0.898	0.652	1.143
Door-to-CT-results	−0.141	0.022	1.941	1	0.107	0.902	0.815	1.227
Constant	9.408	2.355	16.104	1	<0.001 *	11,405.603		

*—Significant association; Cox and Snell R Square = 0.504.

**Table 6:** Logistic regression (using the Enter method) considering thrombolysis as a dependent variable and comorbidities as independent variables.

Variables in the Equation	B	S.E.	Wald	df	Sig.	Exp(B)	95% C.I. for EXP(B)	
							Lower	Upper
**Obesity (Yes)**	**0.122**	**0.716**	**0.029**	**1**	**0.865**	1.129	0.277	4.600
Smoking (Yes)	1.195	0.660	3.283	1	0.070	3.304	0.907	12.037
Dyslipidemia (Yes)	2.664	1.070	6.203	1	0.013 *	14.359	1.764	116.887
Hypertension (Yes)	−2.393	0.667	12.894	1	<0.001 *	0.091	0.025	0.337
Diabetes (Yes)	1.706	0.480	12.632	1	<0.001 *	5.506	2.149	14.105
CHD (Yes)	0.028	0.461	0.004	1	0.951	1.029	0.416	2.540
COPD (Yes)	19.563	4748.850	0.000	1	0.997	313,274,701.511	0.000	
CKD (Yes)	0.808	0.534	2.292	1	0.130	2.243	0.788	6.384
Constant	−25.502	4748.850	0.000	1	0.996	0.000		

*—Significant association; Cox and Snell R Square = 0.482, CHD, chronic heart disease; COPD, chronic obstructive pulmonary disease; CKD, chronic kidney disease.

## Data Availability

The datasets are private, but de-identified data may be provided upon request from Popa Daian.
